# Genetic differentiation of *Plasmodium vivax* duffy binding protein in Ethiopia and comparison with other geographical isolates

**DOI:** 10.1186/s12936-024-04887-1

**Published:** 2024-02-23

**Authors:** Abnet Abebe, Cheikh Cambel Dieng, Sisay Dugassa, Deriba Abera, Tassew T. Shenkutie, Ashenafi Assefa, Didier Menard, Eugenia Lo, Lemu Golassa

**Affiliations:** 1 Aklilu Lemma Institute of Pathobiology, Addis Ababa University, P.O. Box 24756, Addis Ababa, Ethiopia; 2 Ethiopian Public Health Institute, Addis Ababa, Ethiopia; 3 Department of Microbiology and Immunology, College of Medicine, Drexel University, Philadelphia, USA; 4 Institute of Infectious Disease and Global Health, University of North Carolina at Chapel Hill, Chapel Hill, NC USA; 5 Malaria Genetics and Resistance Unit, INSERM U1201, Institut Pasteur, Université Paris Cité, 75015 Paris, France; 6 Dynamics of Host-Pathogen Interactions, Université de Strasbourg, Institute of Parasitology and Tropical Diseases, 67000 Strasbourg, France

**Keywords:** Genetic diversity, *P. vivax*, Duffy Binding Protein, Haplotype network, Ethiopia

## Abstract

**Background:**

*Plasmodium vivax* Duffy binding protein (PvDBP) is a merozoite surface protein located in the micronemes of *P. vivax*. The invasion of human reticulocytes by *P. vivax* merozoites depends on the parasite DBP binding domain engaging Duffy Antigen Receptor for Chemokine (DARC) on these red blood cells (RBCs). PvDBPII shows high genetic diversity which is a major challenge to its use in the development of a vaccine against *vivax* malaria.

**Methods:**

A cross-sectional study was conducted from February 2021 to September 2022 in five study sites across Ethiopia. A total of 58 blood samples confirmed positive for *P. vivax* by polymerase chain reaction (PCR) were included in the study to determine PvDBPII genetic diversity. PvDBPII were amplified using primers designed from reference sequence of *P. vivax* Sal I strain. Assembling of sequences was done using Geneious Prime version 2023.2.1. Alignment and phylogenetic tree constructions using MEGA version 10.1.1. Nucleotide diversity and haplotype diversity were analysed using DnaSP version 6.12.03, and haplotype network was generated with PopART version 1.7.

**Results:**

The mean age of the participants was 25 years, 5 (8.6%) participants were Duffy negatives. From the 58 PvDBPII sequences, seven haplotypes based on nucleotide differences at 8 positions were identified. Nucleotide diversity and haplotype diversity were 0.00267 ± 0.00023 and 0.731 ± 0.036, respectively. Among the five study sites, the highest numbers of haplotypes were identified in Arbaminch with six different haplotypes while only two haplotypes were identified in Gambella. The phylogenetic tree based on PvDBPII revealed that parasites of different study sites shared similar genetic clusters with few exceptions. Globally, a total of 39 haplotypes were identified from 223 PvDBPII sequences representing different geographical isolates obtained from NCBI archive. The nucleotide and haplotype diversity were 0.00373 and 0.845 ± 0.015, respectively. The haplotype prevalence ranged from 0.45% to 27.3%. Two haplotypes were shared among isolates from all geographical areas of the globe.

**Conclusions:**

PvDBPII of the Ethiopian *P. vivax* isolates showed low nucleotide but high haplotype diversity, this pattern of genetic variability suggests that the population may have undergone a recent expansion. Among the Ethiopian *P. vivax* isolates, almost half of the sequences were identical to the Sal-I reference sequence. However, there were unique haplotypes observed in the Ethiopian isolates, which does not share with isolates from other geographical areas. There were two haplotypes that were common among populations across the globe. Categorizing population haplotype frequency can help to determine common haplotypes for designing an effective blood-stage vaccine which will have a significant role for the control and elimination of *P. vivax*.

**Supplementary Information:**

The online version contains supplementary material available at 10.1186/s12936-024-04887-1.

## Background

In 2022, there were an estimated of 6.9 million cases of *Plasmodium vivax* globally [1]. *Plasmodium vivax* malaria is recognized as a cause of severe morbidity and mortality, with a significant negative impact on health in endemic countries [2–4]. The unique biological features of *P. vivax*, such as hypnozoite formation and early gametocytogenesis, pose a challenge to global malaria elimination efforts as traditional control measures may not be as effective. A *P. vivax* vaccine would provide a powerful tool for malaria control [5].

Despite its African origins, the presence of *P. vivax* on the continent is unevenly distributed, with East Africa, particularly the horn of Africa experiencing endemicity and significant clinical disease [6]. In Ethiopia, the impact of *P. vivax* on public health is substantial, given the country’s elevated malaria burden. *P. vivax* constitutes a significant portion of malaria cases, and its transmission occurs seasonally in highland areas where the *Anopheles* mosquito, the primary vector, thrives [7]. This distribution pattern is likely linked to the prevalence of Duffy positive individuals, as *P. vivax* is believed to rely on the Duffy receptor for invasion of reticulocytes and the manifestation of clinical disease. These findings underscore the significance of studying *P. vivax* in Ethiopia, emphasizing its prevalence and the need for further research in this context.

The invasion pathway of *P. vivax* into reticulocytes was previously believed to primarily depend on the interaction between the Duffy binding protein (PvDBP) and its receptor, the Duffy Antigen Receptor for Chemokines (DARC) [8–10]. *P. vivax* Duffy binding protein (PvDBP) is a merozoite surface protein of 140 kDa located in the micronemes of *P. vivax*, and is used to invade RBCs [11]. The erythrocyte-binding motif of DBP resides in the N-terminal conserved cysteine-rich domain (C1–C12) known as DBP region II [12–14]. Region II of PvDBP1 (PvDBPII) is 330 amino acids in length and contains 12 cysteines that are conserved among different DBL domains. Since it has conserved cysteine-rich domain, PvDBPII is one of the most promising vaccine targets [15, 16]. The central stretch of *P. vivax* region II (cysteines at positions 4–7) is important for receptor recognition and critical for binding to DARCs on human RBCs [15, 17].

Genetic variation analysis indicated that PvDBPII shows high genetic diversity [18] and has a 4 times higher substitution rate compared to the rest of the gene [19, 20]. The region involved in RBC binding is highly polymorphic and there is evidence of strain specific protection [21]. The current vaccine development is based on the DBPII haplotype of the *P. vivax* laboratory-adapted strain Sal-1 [11]. So identifying the genetic diversity in PvDBPII and its dominant haplotypes is important for the rational design of DBP-based vaccine against *vivax* malaria. This study aims to assess the genetic diversity of PvDBPII across various study sites in Ethiopia and comparison with isolates from other regions of the world.

## Methods

### Study sites and sample collection

**Fig. 1:**
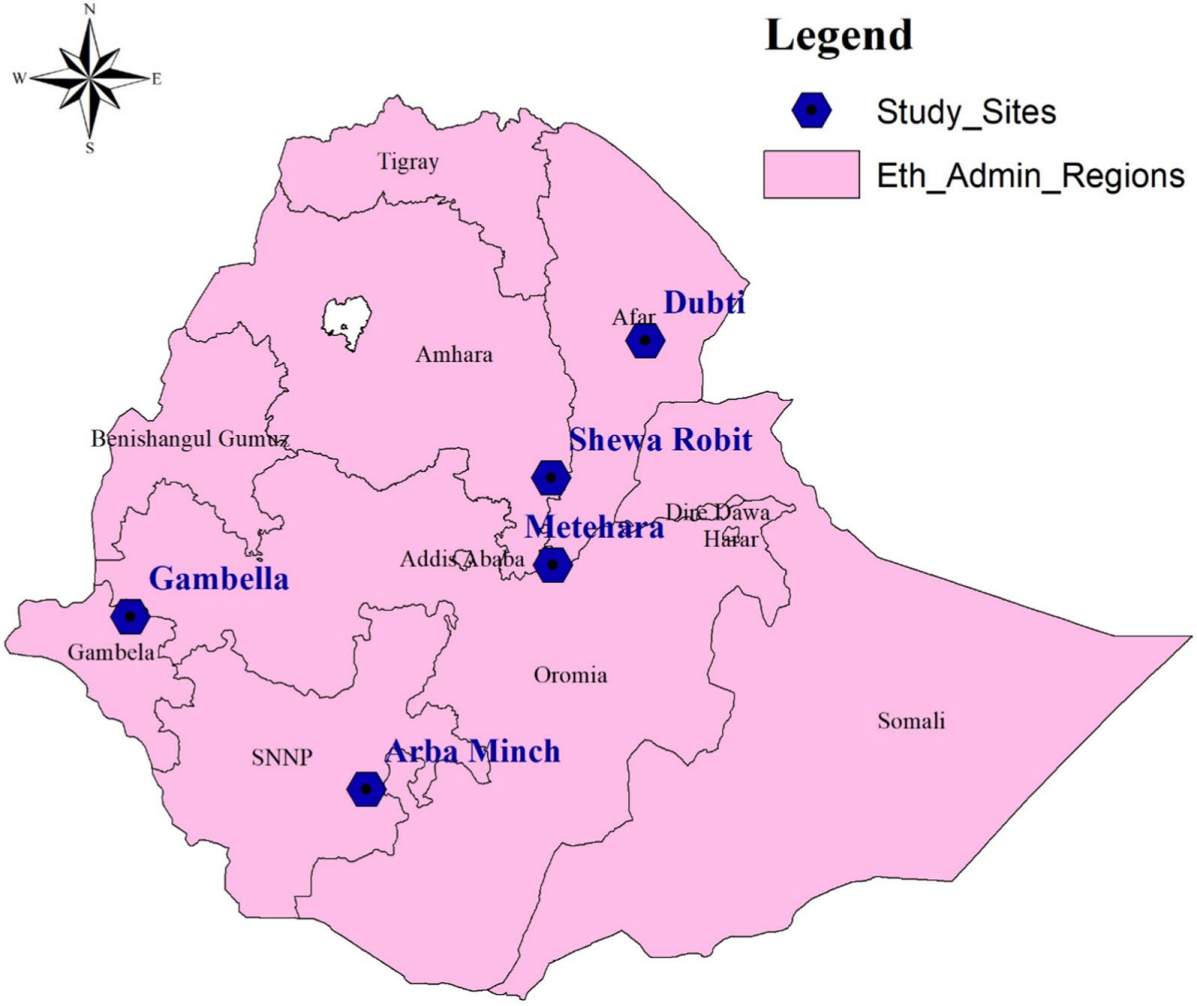
A map of the study sites, Ethiopia

A health-facility based cross-sectional study was conducted from February 2021 to September 2022 at five hospitals: Arbaminch, Dubti, Gambella, Metehara, and Shewarobit hospitals (Fig. 1). Finger prick blood sample was collected from each malaria suspected febrile individuals for the purpose of doing malaria laboratory diagnosis using microscopy and Rapid Diagnostic Tests (RDTs). In order to maximize the accuracy of research participant inclusion, patients who were diagnosed positive for *P. vivax* by both RDT and microscopy were included in the study. Parasite densities were determined on thick blood film by malaria microscopists by considering a minimum of 200 WBCs, the reported number of parasites per microliter of blood, and an assumed total white blood cell count of 8000/μL. Venous blood samples were collected and spotted onto Whatman 903 Protein Saver Card, US (Dried Blood Spot, DBS). Genomic DNA (parasite and human DNA) was extracted from a 6 mm diameter spot of a DBS sample and eluted in 100 µl of TE Buffer (Tris–EDTA) using the QIAamp DNA Extraction Kit according to the manufacturer’s instructions. To avoid contamination, the scissor blade (puncher) and forceps were immersed in the RNASE AWAY solution for 1 min and then cleaned with distilled water in between each sample. In addition to the microscopy and RDT, malaria parasites identification was confirmed by Bio-Rad CFX96 Real-Time PCR [22].

### Amplifications and sequencing of PvDBPII

*P. vivax* samples collected at five hospitals were evaluated for PvDBPII polymorphism. The amplification of PvDBP sequences utilized primers targeting the DBP1 region II, (forward: 5′-GATATTGATCATAAGAAAACGATCTCTAGT- 3′; reverse: 5′-TGTCACAACTTCCTGAGTATTTTTTTTAGCCTC- 3′) [23], designed based on the reference sequence *P. vivax* PVX_110810 from of Sal I (Reference assembly: NC_009911.1). The amplification was carried out in a 20-μL reaction mixture containing 2 μL of genomic DNA, 10 μL of 2 × DreamTaq Green PCR Master Mix (Fermentas), and 0.5 μM primer. Amplification reactions were performed in a Bio-Rad T100 thermal cycler, with an initial denaturation at 94 °C for 2 min, followed by 35 cycles at 94 °C for 30 s, 58 °C for 30 s, and 72 °C for 90 s with a final 5-min extension at 72 °C. PCR products were purified prior to Sanger sequencing using Sephadex G-50 (Merck, Darmstadt, Germany) and purified amplicons were then used as templates for cycle sequencing reaction, using the Applied Biosystems BigDye Terminator v3.1 Cycle Sequencing Kit, according to manufacturers’ protocols. Sequences were detected on the SeqStudio genetic analyzer (Applied Biosystems, California, USA) and the data were generated as Fasta format for analysis. In order to ensure quality, positive and negative controls were used in each PCR run, and gel electrophoresis was performed to visualize the amplified product.

### Genetic diversity and statistical analysis

Sequences of PvDBPII genes were trimmed and assembled using De Novo assembler of Geneious Prime version 2023.2.1 [24]. Pairwise and multiple aliment was done using ClustalW alignment algorithm in Diversity Molecular evolutionary Genetic Analysis (MEGA X). Missing values across the column of the total sequences were removed using Jalview version 2.11.2 (a multiple sequence alignment editor and analysis workbench) [25]. All ambiguous positions were removed for each sequence pair (pairwise deletion option). Nucleotide substitution (r) pattern and rates were estimated under the Tamura-Nei model, and phylogenetic tree was built to see evolutionary divergence between Sequences using MEGA version 10.1.1 software [26]. Nucleotide diversity (π), number of segregating sites (S), the number of haplotypes (H), haplotype diversity (Hd), the total number of mutations (η), and the average number of nucleotide differences (k) were analysed using DnaSP version 6.12.03 software [27]. Frequencies were analysed using Statistical Package for Social Sciences (SPSS, version 25). P-values less than 0.05 were considered as statistically significant.

### Genetic differentiation and haplotype network analysis

Gene flow and genetic differentiation estimates (Fst) were analysed using DnaSP version 6.12.03 software [27] and haplotypes network was constructed using PopART version 1.7 software [28]. The level of genetic differentiation of PvDBPII was estimated by *F*_ST_ values. These values were interpreted based on established criteria: 0–0.05 indicating minimal genetic differentiation, 0.05–0.15 denoting moderate differentiation, 0.15–0.25 representing substantial differentiation, and values exceeding 0.25 signifying significant genetic divergence [29]. To estimate the degree of genetic differentiation of the PvDBPII in global isolates, a total of 165 global PvDBPII sequences from 5 *P. vivax* endemic countries of different continents were retrieved from the National Centre for Biotechnology Information (NCBI) archive. These included; isolates from South America (n = 34): Brazil (EU812840.1-EU812873.1) [30], isolates from Asia (n = 9): Iran (EU860429-EU860437) [31], isolates from North America (n = 35): Mexico (KP759780-KP759814) [32], isolates from Oceania (n = 45): Papua New Guinea (PNG) (AF469550:AF469594) [33], and 42 isolates from Africa: Sudan (MG805616.1-MG805657.1) [13]. The reference sequence from Sal-1 (NC_009911.1)) was included in the final analysis.

## Results

### Characteristics of the study participants

A total of 80 samples with 16 samples per study site were sequenced to assess genetic diversity of PvDBP. Of these, 58 isolates which were effectively sequenced and had good quality were considered for analysis. The mean parasite density of the asexual stage was 9735 parasite/µl. Out of the 58 participants, 38 (65.5%) were male, the mean age was 25 years, and the study participants aged between 20 and 40 years [18] accounted for 70.7% (41)(Table 1). The frequency of male participants from ArbaMinch, Shewarobit, Metehara, Gambella and Dubti was 9/11 (81.8%), 4/7 (57.1%), 9/14 (64.3%), 6/12 (50.0%), and 10/14 (71.4%), respectively.

**Table 1:** Demographic information of study participants

Characteristics	Gender		Study Sites					Age			Total
	Female	Male	Arbaminch	ShewaRobit	Metehara	Gambella	Dubti	< 20	20–40	> 40	
Frequency	20	38	11	7	14	12	14	15	41	2	58
Percentage (%)	34.5	65.5	19.0	12.1	24.1	20.7	24.1	25.9	70.7	3.4	100

### Nucleotides and amino acid frequencies of PvDBPII gene sequences

Analysis was done using the consensus sequence of 736 bps which corresponds to nucleotide positions from 838 to 1573 of the Salvador I reference. The nucleotide frequencies of the 58 sequences were 40.36% (A), 26.06% (T), 11.66% (C), and 21.92% (G). The mean percent of GC in assembled sequence was 33.5%. The amino acid with highest frequency was leucine (L) (9.11) followed by alanine (A) 97.69%, whereas the frequency of tryptophan (W) (1.43%) was the least (Table 2).

**Table 2:** The amino acid frequencies among the 58 sequences

Amino acid	A	R	N	D	C	Q	E	G	H	I	L	K	M	F	P	S	T	W	Y	V
Frequency (%)	7.69	5.11	4.25	5.13	2.03	4.11	6.18	7.47	2.30	5.26	9.11	5.95	2.34	4.05	5.05	6.82	5.85	1.43	3.23	6.64

A: alanine; R: arginine; N: asparagine; D: aspartic acid; C: cysteine; Q: glutamine; E: glutamic acid; G: glycine; H: histidine; I: isoleucine; L: leucine; K: lysine; M: methionine; F: phenylalanine; P: proline; S: serine; T: threonine; W: tryptophan, Y: tyrosine, V: valine

### Single nucleotide polymorphism (SNP) of PvDBPII among the Ethiopian isolates

**Fig. 2:**
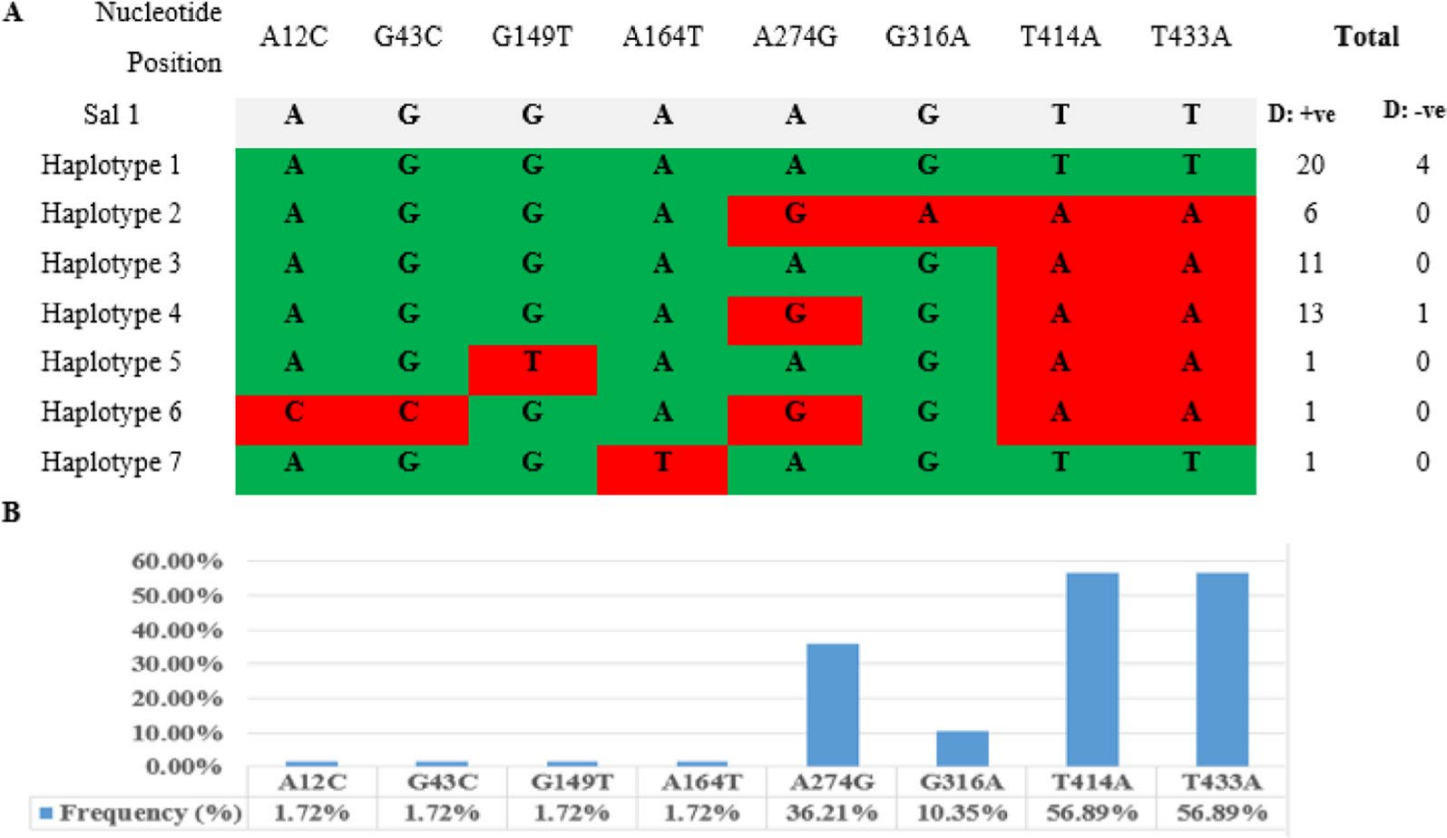
Single nucleotides polymorphism of PvDBPII among Ethiopian isolates. **A** Position of nucleotide changes. Polymorphic nucleotides are listed for each haplotype. Nucleotides identical to those of the reference sequence, Sal-I (NC_009911.1), are marked in green. The dimorphic nucleotide changes are marked in red color. Y-axis represent haplotypes list in the left while the total number of sequences for each haplotype at right panel (D: + ve; Duffy positive, and D:-ve; Duffy negative). X-axis represent nucleotides position. **B** Frequencies (%) of nucleotide changes found in PvDBPII among 58 Ethiopian isolates. Frequencies in percent denoted on the Y-axis, and nucleotide changes on the X-axis

The number of segregating sites were 8, with a nucleotide diversity of 0.00267 ± 0.00023 and an average of 1.791 pairwise nucleotide differences (k). The nucleotide diversity per study site was 0.00295, 0.00177, 0.00216, 0.00363, and 0.0022 among the isolates from Metehara, Gambella, Dubti, ArbaMinch, and ShewaRobit, respectively (Table 3). A total of 8 mutations were observed, consisting of 4 singleton mutations (present in only one sequence) at positions 12, 43, 149, and 164, and 4 parsimony informative sites (with at least two types of nucleotides or occurring with a minimum frequency of two) at positions 274, 316, 414, and 433. From the 58 PvDBPII sequences, 7 haplotypes were identified (GenBank accession numbers: PP141174-PP141180), yielding a haplotype diversity of 0.731 ± 0.036. Among the 58 *P. vivax* isolates from Ethiopia, 41.38% (24 sequences) matched the Sal-1 reference sequence, with the majority originating from Gambella (37.5% of the 24). All identified haplotypes exhibited dimorphism, except for the predominant haplotype 1, which accounted for 41.38% (24 out of 58) of the Ethiopian isolates (Fig. 2). Of the total sequences, the Duffy status of five isolates were negative, and of these most of them (80% of the 5) were identified as haplotype 1 and the other one was haplotype 4 (see Additional file 1).

**Table 3:** Nucleotide and haplotype diversity of PvDBPII gene and number of isolates per haplotypes among different Ethiopian populations

Sites	N	S	π	H	Hd	Number of sequences/H						
						H1	H2	H3	H4	H5	H6	H7
Metehara	14	4	0.00295	3	0.6923	5	6	3	0	0	0	0
Gambella	12	3	0.00177	2	0.4091	9	0	0	3	0	0	0
Dubti	14	3	0.00216	3	0.7033	6	0	4	4	0	0	0
ArbaMinch	11	7	0.00363	6	0.8545	2	0	2	4	1	1	1
ShewaRobit	7	3	0.0022	3	0.7619	2	0	2	3	0	0	0
Total	58	8	0.00267	7	0.731	24	6	11	14	1	1	1

N: number of isolates; S: Number of segregating (polymorphic/variable) sites; H, Number of haplotypes; Hd: Haplotype diversity; π: nucleotide diversity

### Diversity and evolutionary history among isolates from different study sites

Within the five study sites, Arbaminch exhibited the highest diversity, housing 6 distinct haplotypes, while Gambella had only 2. Haplotype 2 was exclusive to Metehara, while Arbaminch contained haplotypes 5, 6, and 7. Notably, three haplotypes (5.17%) were represented by a single sequence. The nucleotide diversity of PvDBPII in Arbaminch (π = 0.00363) surpassed that of the other sites (Table 3).

**Fig. 3:**
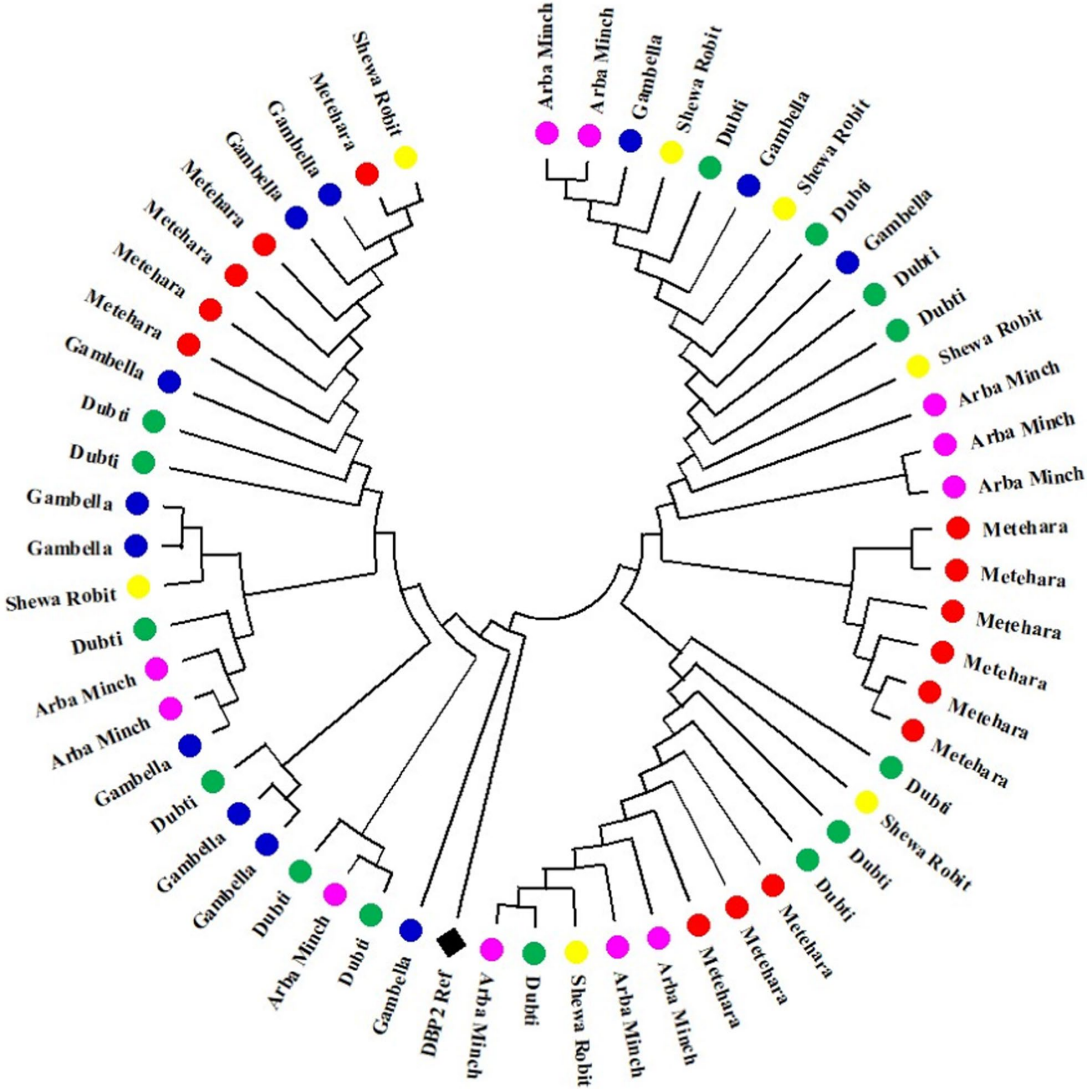
Neighbour-Joining phylogenetic tree based on PvDBP-II-gene SNPs of Arbaminch (n = 11), Gambella (n = 12), Metehara (n = 14), Dubti (n = 14) and Shewarobit (n = 7) isolates

The Neighbour-Joining method was employed to infer the evolutionary history, with evolutionary distances calculated using the Tajima-Nei method, measured in the number of base substitutions per site. The phylogenetic tree, based on PvDBPII SNPs, indicated a shared profile among parasites from different study sites, with a few exceptions. Notably, the majority of isolates from Gambella (9 out of 12, or 75%) exhibited a close relationship with the Sal-1 reference strain, while isolates from Arbaminch showed the least relationship (3 out of 11, or 27%) (Fig. 3).

### Genetic differentiation and haplotypes network among global isolates

A total of 39 haplotypes were identified from 223 PvDBPII gene sequences, consisting of 58 sequences from Ethiopia and 165 obtained from the NCBI archive (global isolates). The average number of nucleotide differences (k) was 2.411, with 40 segregating sites, a nucleotide diversity of 0.00373, and a haplotype diversity of 0.845 ± 0.015. The genetic differentiation between Ethiopian isolates and those from Iran was minimal, as indicated by an F_ST_ value of 0.00. However, there was moderate differentiation between Ethiopian isolates and those from Sudan, with an FST value of 0.0697. Ethiopian isolates were most differentiated from PNG isolates (0.1846). Notably, isolates from Papua New Guinea exhibited significant genetic differentiation from those in Sudan and Mexico, with FST values of 0.3051 and 0.3336, respectively (Table 4).

**Table 4:** Nucleotide and haplotype diversity of PvDBPII gene and Fst of global populations

Population	π	H	Hd	*F*_ST_					
				BR	ET	IR	ME	PNG	SU
Brazil (n = 34)	0.00402	8	0.811	–					
Ethiopia (n = 58)	0.00267	7	0.731	0.1115	–				
Iran (n = 9)	0.00361	5	0.861	0.00966	0.00	–			
Mexico (n = 35)	0.00161	4	0.501	0.2804	0.1263	0.1715	–		
PNG (n = 45)	0.00478	20	0.861	0.1191	0.1846	0.1062	0.3336	–	
Sudan (n = 42)	0.00255	10	0.841	0.2526	0.0697	0.1398	0.0454	0.3051	–
Total (n = 223)	0.00373	39	0.845						

Populations from Brazil (BR), Ethiopia (ET), Iran (IR), Mexico (ME),Papua New Guinea (PNG) and Sudan (SU). n; number of isolates, H; Number of haplotypes, Hd; Haplotype diversity; π; nucleotide diversity, *F*_ST_; Fixation index, a measure of genetic differentiation between populations

**Fig. 4:**
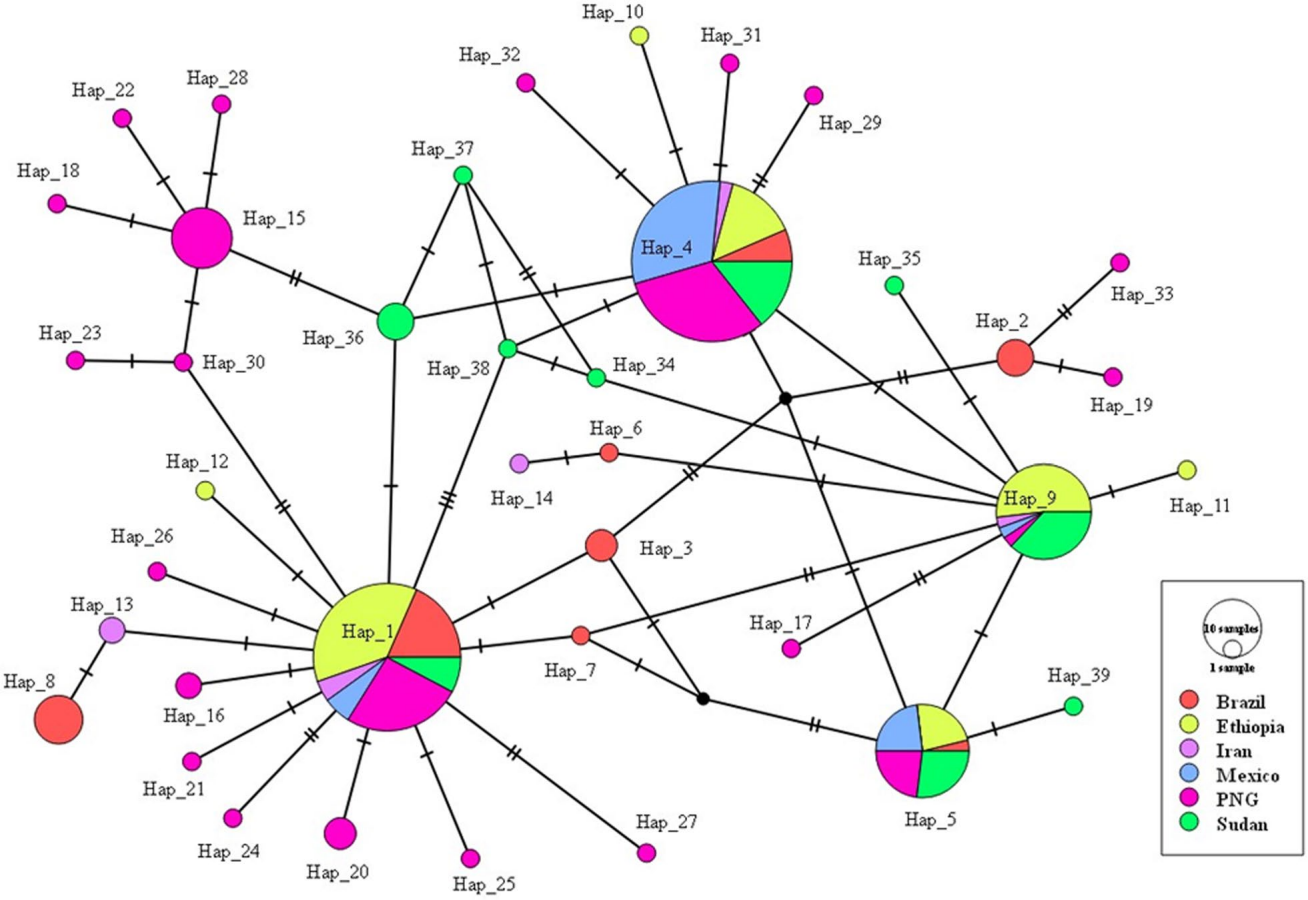
Networking of PvDBPII obtaining from Ethiopian P. vivax isolates and isolates from different malaria endemic countries; Brazil, Iran, Mexico, Papua New Guinea, and Sudan

In the network analysis, the haplotype prevalence ranged from 0.45% to 27.3%. Both haplotypes H1 (Sal-1 like) and H4 were present across isolates from all geographical areas of the globe. Haplotypes H5 and H9 were shared among all geographic locations except those from Iran and Brazil, respectively. The most prevalent haplotype H1 (27.3%) was shared with multiple populations followed by H4 (23.8%). Twenty-six haplotypes (66.67%) were represented by a single sequence. Three haplotypes (H10, H11, and H12) were exclusive to Ethiopian isolates, not shared with those from other geographical areas. The network comprised a total of 48 connections among haplotypes, with 36 (75%) differing by one nucleotide, and 12 (25%) varying by two nucleotides (Fig. 4).

## Discussion

The analysis of PvDBPII gene sequences from diverse geographical regions sheds light on several noteworthy patterns of parasite evolution and implications, such as evaluation of malaria vaccine candidates [34]. The range of observed haplotype prevalence, spanning from 0.45% to 27.3%, serves as an indicator of the inherent genetic diversity within *P. vivax* populations. This observation is particularly relevant considering that the ongoing vaccine development is solely grounded in the DBPII haplotype of the laboratory-adapted *P. vivax* strain Sal-1 [11]. This study was the first to report polymorphism on the isolates from five different malaria endemic sites in the country. PvDBPII polymorphisms were investigated in 58 *P. vivax* isolates from endemic study sites to expand the knowledge on PvDBPII gene population diversity in Ethiopia and compare the data with those from other endemic countries in the world. In general, this study identified 7 haplotypes from Ethiopian isolates and 39 haplotypes from the global isolates.

Parasite genetic diversity has been considered a potential indicator of transmission intensity [35, 36], particularly in areas nearing malaria elimination where routine surveillance and case reporting may be less reliable [35]. In examining the molecular polymorphism within the 58 PvDBPII sequences, revealed a relatively lower nucleotide diversity (π = 0.00267 ± 0.00023) compared to documented studies in various regions. Notably, the nucleotide diversity observed in this study was lower than findings in Mexico (π = 0.00304) [32], Sudan (π = 0.00497) [13], China (π = 0.0059) [37], Ethiopia (π = 0.00608) [38], Ecuador (0.00736) [39], Brazil (π = 0.0081) [30], and Thailand (π = 0.012) [35]. These variations underscore the dynamics of malaria transmission intensity and emphasize the importance of region-specific assessments in understanding the genetic landscape of *P. vivax* [35]. The overall haplotype diversity observed in Ethiopia was 0.731, surpassing the reported diversity in Mexico (0.556) [32], and aligning closely with isolates from the Amazon region, Brazil (0.715) [40]. However, it was relatively lower than the haplotype diversity documented in Thailand (0.849) [35], Eastern Myanmar (0.850) [41], Ethiopia (0.874) [38], Sudan (0.900), China (0.91) [37], Sri Lanka (0.922) [34], Brazil (0.934) [30], Ecuador (0.940) [39], and Brazil (0.98) [42]. These comparative diversity measures underscore the unique genetic landscape of *P. vivax* populations and highlight the importance of regional context in understanding haplotype diversity [37].

In this study, seven haplotypes were identified from a pool of 58 PvDBPII sequences. This represents a relatively low number of haplotypes compared to a prior study in Ethiopia where nine distinct haplotypes were discerned from 23 isolates [38]. Although the phylogenetic tree based on PvDBPII SNPs indicated shared profiles among parasites from different study sites, with few exceptions, the haplotype diversity varied across sites, ranging from 0.8545 in Arbaminch to 0.4091 in Gambella. This may be due to the difference in transmission intensity of *P. vivax* at different part of the country [35]. Among the 58 Ethiopian sequences, the highest number which was 24 (41.4%) sequences were identical to Sal-1 reference sequence and shared with multiple populations. The identification of haplotypes identical to the Sal-1 reference sequence, and those shared across populations, may hold significance for vaccine development [37]. The DBPII Sal-1 variant is currently utilized in the development of a PvDBP-based vaccine, making these haplotypes potentially attractive candidates for further vaccine research and development [40].

For development of PvDBP-based vaccine it is important to assess the levels of DBPII genetic diversity among *P. vivax* field isolates globally [11]. Comparing the genetic differentiation among isolates from different regions provides insights into the evolutionary dynamics of *P. vivax*. The negligible genetic differentiation between Ethiopian isolates and those from Iran suggests a degree of gene flow, shared ancestry or stronger functional constraints on PvDBPII [35]. In contrast, moderate differentiation with isolates from Sudan indicates regional distinctions in parasite populations. Notably, isolates from Papua New Guinea display substantial genetic differentiation from those in Sudan and Mexico, suggesting distinct evolutionary trajectories in these regions and or the geographic barrier between the countries inhibiting gene flow [38].

The haplotype network configuration of PvDBPII provided the information about intragenic recombination in these gene fragments. Network analysis further elucidates the relationships among haplotypes, revealing both single and multiple nucleotide variations. A haplotype network was constructed to establish the relationships among the PvDBPII haplotypes from the 223 isolates of six malaria endemic countries from different continents in the world. A total of 39 haplotypes were identified with haplotype prevalence ranging from 0.45% to 27.3%. Such a finding advanced the knowledge on the parasite population dynamics in this country for the rational design of effective interventions to block disease transmission [37].There were three haplotypes only with the Ethiopian isolates, which does not shared with isolates of different geographical areas. Existing of such unique geographical sequences, may be due to biogeographic limitations, or may be due to the possible recent introduction of the parasite to that geographic area [34].

## Conclusion

This study highlights the intricate genetic landscape of PvDBPII in *P. vivax* populations in Ethiopia and globally. The prevalence and distribution of haplotypes, regional variations, and genetic differentiation underscore the complex interplay of factors shaping the evolutionary dynamics of this parasite. Understanding these dynamics is crucial for tailoring effective malaria control strategies and advancing the knowledge on *P. vivax* biology. Such insights enable investigators to select dominant haplotypes crucial for designing an effective blood-stage vaccine thereby playing a pivotal role in the control and elimination of *P. vivax*.

### Supplementary Information

**Additional file 1:** **Figure S1.** **A** Conting view of consensus sequence coverage of Ethiopian isolates against the referance sequence. **B **Consensus sequence coverage of Global isolates (223 sequences and referance).

## Data Availability

All relevant data are within the manuscript. The data that support the findings of this study are available from the corresponding author on reasonable request.

## Abbreviations

BPs: Base Pairs
DARC: Duffy Antigen Receptor for Chemokine
DBS: Dried blood spots
DNA: Deoxyribonucleic acid
*F*_ST_: Fixation Index
Hd: Haplotype diversity
MEGA: Diversity Molecular evolutionary Genetic Analysis
NCBI: National Centre for Biotechnology Information
*Pv/Pf*: *Plasmodium* *vivax* and *Plasmodium* *falciparum*
PvDBP: *P. vivax* Duffy Binding Protein
RBCs: Red blood cells
RDT: Rapid diagnostic tests
RT PCR: Real Time Polymerase Chain Reaction
SNP: Single nucleotide polymorphism
SPSS: Statistical package for social sciences
