# Supplementary material for: Genetic differentiation of Plasmodium vivax duffy binding protein in Ethiopia and comparison with other geographical isolates
Source: Malar J. 2024 Feb 23;23:55. doi: 10.1186/s12936-024-04887-1 (PMC10885561; doi:10.1186/s12936-024-04887-1)
Supplement: Supplementary file 1 — Additional file 1: Figure S1. A Conting view of consensus sequence coverage of Ethiopian isolates against the referance sequence. B Consensus sequence coverage of Global isolates (223 sequences and referance). [file 12936_2024_4887_MOESM1_ESM.docx]

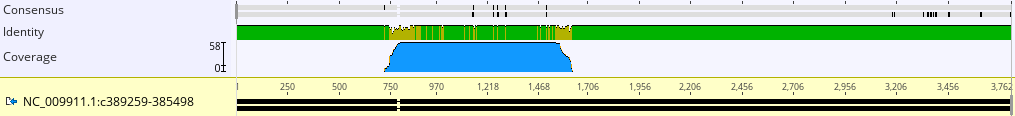


**Fig. S1A**: Conting view of consensus sequence coverage of Ethiopian isolates against the referance sequence


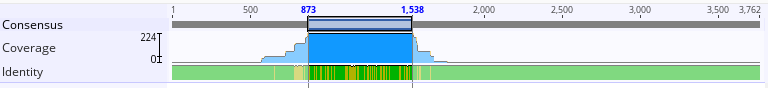


**Fig. S1B**: Consensus sequence coverage of Global isolates (223 sequences and referance)
